# Supplementary material for: Fetal Growth Restriction: Comparison of Biometric Parameters
Source: J Pers Med. 2022 Jul 11;12(7):1125. doi: 10.3390/jpm12071125 (PMC9317726; doi:10.3390/jpm12071125)
Supplement: Supplementary file 1 [file jpm-12-01125-s001.zip › Supplements.pdf]

Table S1: Regression results for percentile plots of sonographic parameters. Model fit was performed for 10th, 50th and 90th percentile for each sonographic ratio fitted by: ratio ~ **a+b*GA+c*GA²+d*GA³**. AC = abdominal circumference, BPD = biparietal diameter, CI = confidence interval, FL = femur length, HC = head circumference, pct = percentile, TCD = transcerebellar diameter.

Ratio	Estimate	95% CI	*p* value
**HC (10th pct)**			
a	-5.5328	-21.4299 – 10.3644	0.4951
b	2.1830	0.0809 – 4.2852	0.0418
c	0.4936	0.4037 – 0.5836	<.0001
d	-0.0086	-0.0098 – -0.0073	<.0001
**HC (50th pct)**			
a	-3.9716	-17.7150 – 9.7719	0.5711
b	2.1841	0.4173 – 3.9508	0.0154
c	0.5179	0.4441 – 0.5917	<.0001
d	-0.0090	-0.0100 – -0.0080	<.0001
**HC (90th pct)**			
a	13.2957	-5.5018 – 32.0932	0.1656
b	0.3035	-2.1751 – 2.7821	0.8103
c	0.6126	0.5066 – 0.7186	<.0001
d	-0.0102	-0.0117 – -0.0088	<.0001
**BPD (10th pct)**			
a	8.3921	2.5741 – 14.2102	0.0047
b	-0.4590	-1.2274 – 0.3093	0.2416
c	0.1721	0.1394 – 0.2048	<.0001
d	-0.0027	-0.0032 – -0.0023	<.0001
**BPD (50th pct)**			
a	2.2356	-2.8324 – 7.3037	0.3872
b	0.3997	-0.2703 – 1.0696	0.2423
c	0.1463	0.1175 – 0.1751	<.0001
d	-0.0024	-0.0028 – -0.0021	<.0001
**BPD (90th pct)**			
a	6.7349	0.7516 – 12.7182	0.0274
b	-0.0910	-0.8707 – 0.6888	0.8191
c	0.1743	0.1413 – 0.2073	<.0001
d	-0.0028	-0.0033 – -0.0024	<.0001
**AC (10th pct)**			
a	-69.0745	-87.7419 – -50.4071	<.0001
b	9.5145	7.0579 – 11.9711	<.0001
c	0.0936	-0.0110 – 0.1983	0.0794
d	-0.0020	-0.0034 – -0.0006	0.0061
**AC (50th pct)**			
a	-70.9656	-84.3577 – -57.5735	<.0001
b	10.2846	8.5417 – 12.0275	<.0001
c	0.0727	-0.0011 – 0.1465	0.0535
d	-0.0016	-0.0026 – -0.0006	0.0022
**AC (90th pct)**			
a	-46.3402	-68.7357 – -23.9446	<.0001
b	7.4990	4.5507 – 10.4472	<.0001
c	0.2056	0.0804 – 0.3309	0.0013
d	-0.0033	-0.0050 – -0.0016	0.0002
**FL (10th pct)**			

a	-39.8647	-44.1893 – -35.5401	<.0001
b	4.2383	3.6761 – 4.8005	<.0001
c	-0.0348	-0.0583 – -0.0112	0.0038
d	0.0000	-0.0004 – 0.0003	0.8293
**FL (50th pct)**			
a	-40.2490	-43.2771 – -37,2210	<.0001
b	4.4077	4.0241 – 4.7912	<.0001
c	-0.0385	-0.0544 – -0.0227	<.0001
d	0.0000	-0.0002 – 0.0002	0.8453
**FL (90th pct)**			
a	-29.7584	-33.4226 – -26.0942	<.0001
b	3.2440	2.7802 – 3.7078	<.0001
c	0.0114	-0.0077 – 0.0305	0.2405
d	-0.0006	-0.0009 – -0.0004	<.0001
**TCD (10th pct)**			
a	16.3571	11.5841 – 21.1301	<.0001
b	-1.5250	-2.1720 – -0.8780	<.0001
c	0.1092	0.0809 – 0.1376	<.0001
d	-0.0013	-0.0017 – -0,0009	<.0001
**TCD (50th pct)**			
a	17.1858	13.3614 – 21.0101	<.0001
b	-1.4903	-2.0216 – -0.9590	<.0001
c	0.1048	0.0809 – 0.1287	<.0001
d	-0.0011	-0.0014 – -0,0008	<.0001
**TCD (90th pct)**			
a	18.3239	12.7680 – 23.8798	<.0001
b	-1.4625	-2.2075 – -0.7176	<.0001
c	0.1004	0.0680 – 0.1327	<.0001
d	-0.0009	-0.0014 – -0,0005	<.0001
**FL/AC (10th pct)**			
a	-0.1660	-0.1934 – -0.1386	<.0001
b	0.0385	0.0352 – 0.0418	<.0001
c	-0.0013	-0.0014 – -0,0012	<.0001
d	0.0000	0.0000 – 0.0000	<.0001
**FL/AC (50th pct)**			
a	-0.1325	-0.1544 – -0,1106	<.0001
b	0.0364	0.0337 – 0.0391	<.0001
c	-0.0012	-0.0013 – -0,0011	<.0001
d	0.0000	0.0000 – 0.0000	<.0001
**FL/AC (90th pct)**			
a	-0.0662	-0.1013 – -0,0311	0.0002
b	0.0308	0.0266 – 0.0351	<.0001
c	-0.0010	-0.0012 – -0,0008	<.0001
d	0.0000	0.0000 – 0.0000	<.0001
**TCD/AC (10th pct)**			
a	34.1176	31.3786 – 36.8566	<.0001
b	-2.3949	-2.7367 – -2.0530	<.0001
c	0.0853	0.0713 – 0.0993	<.0001
d	-0.0010	-0.0012 – -0.0008	<.0001
**TCD/AC (50th pct)**			
a	39.1917	37.5438 – 40.8396	<.0001
b	-2.8566	-3.0659 – -2.6473	<.0001

c	0.1018	0.0931 – 0.1104	<.0001
d	-0,0011	-0.0013 – -0.0010	<.0001
**TCD/AC (90th pct)**			
a	45.2301	42.3682 – 48.0919	<.0001
b	-3.3964	-3.7590 – -3.0337	<.0001
c	0.1200	0.1050 – 0.1350	<.0001
d	-0.0013	-0.0015 – -0.0011	<.0001
**HC/AC (10th pct)**			
a	15.3218	14.1559 – 16.4878	<.0001
b	-0.4693	-0.6111 – -0.3276	<.0001
c	0.0172	0.0116 – 0.0228	<.0001
d	-0.0002	-0.0003 – -0.0002	<.0001
**HC/AC (50th pct)**			
a	18.2726	17.1183 – 19.4270	<.0001
b	-0.7345	-0.8759 – -0.5932	<.0001
c	0.0272	0.0216 – 0.0328	<.0001
d	-0.0004	-0.0004 – -0.0003	<.0001
**HC/AC (90th pct)**			
a	18.5081	16.8963 – 20.1200	<.0001
b	-0.6491	-0.8422 – -0.4559	<.0001
c	0.0231	0.0156 – 0.0305	<.0001
d	-0.0003	-0.0004 – -0.0002	<.0001

Table S2: Regression results for percentile plots of sonographic parameters. Model fit was performed for 3th and 97th percentile for each sonographic ratio fitted by: ratio ~ **a+b*GA+c*GA²+d*GA³**. AC = abdominal circumference, BPD = biparietal diameter, CI = confidence interval, FL = femur length, HC = head circumference, pct=percentile, TCD = transcerebellar diameter.

ratio	Estimate	95% CI	*p* value
**HC (3th pct)**			
a	-10.2006	-33.4466 – 13.0455	0.3897
b	2.5757	-0.4888 – 5.6403	0.0995
c	0.4719	0.3415 – 0.6024	<.0001
d	-0.0083	-0.0101 – -0.0065	<.0001
**HC (97th pct)**			
a	16.2585	-10.0805 – 42.5975	0.2263
b	0.1701	-3.3262 – 3.6665	0.9240
c	0.6214	0.4706 – 0.7722	<.0001
d	-0.0103	-0.0124 – -0.0082	<.0001
**BPD (3th pct)**			
a	13.4294	4.4180 – 22.4407	0.0035
b	-1.1306	-2.2904 – 0.0292	0.0561
c	0.1943	0.1461 – 0.2425	<.0001
d	-0.0030	-0.0036 – -0.0023	<.0001
**BPD (97th pct)**			
a	12.7560	3.4725 – 22.0395	0.0071
b	-0.7646	-1.9337 – 0.4046	0.1999
c	0.2035	0.1560 – 0.2511	<.0001
d	-0.0032	-0.0038 – -0.0026	<.0001
**AC (3th pct)**			
a	-63.2831	-88.4957 – -38.0704	<.0001
b	8.6451	5.3611 – 11.9292	<.0001
c	0.1172	-0.0197 – 0.2542	0.0933
d	-0.0023	-0.0041 – -0.0004	0.0147
**AC (97th pct)**			
a	-33.1537	-63.8501 – -2.4573	0.0343
b	5.9026	2.0297 – 9.7755	0.0028
c	0.2862	0.1275 – 0.4448	0.0004
d	-0.0044	-0.0065 – -0.0023	<.0001
**FL (3th pct)**			
a	-37.3809	-42.98931 – -31.7686	<.0001
b	3.9113	3.2008 – 4.6218	<.0001
c	-0.0243	-0.0536 – 0.0050	0.1035
d	-0.0002	-0.0005 – 0.0002	0.4323
**FL (97th pct)**			
a	-25.7335	-32.4664 – -19.0006	<.0001
b	2.7815	1.9403 – 3.6227	<.0001
c	0.0329	-0.0013 – 0.0670	0.0593
d	-0.0009	-0.0014 – -0.0005	<.0001
**TCD (3th pct)**			
a	7.4550	0.2833 – 14.6268	0.0416
b	-0.4535	-1.4037 – 0.4968	0.3496
c	0.0666	0.0259 – 0.1073	0.0013
d	-0.0008	-0.0014 – -0.0002	0.0049
**TCD (97th pct)**			

a	20.8028	6.7502 – 34.8553	0.0037
b	-1.7493	-3.7387 – 0.2400	0.0848
c	0.1122	0.0209 – 0.2035	0.0160
d	-0.0010	-0.0024 – 0.0003	0.1384
**FL/AC (3th pct)**			
a	-0.1938	-0.2343 – -0.1532	<.0001
b	0.0412	0.0362 – 0.0463	<.0001
c	-0.0014	-0.0016 – -0.0012	<.0001
d	0.0000	0.0000 – 0.0000	<.0001
**FL/AC (97th pct)**			
a	-0.0506	-0.1246 – 0.0234	0.1804
b	0.0299	0.0208 – 0.0389	<.0001
c	-0.0010	-0.0013 – -0.0006	<.0001
d	0.0000	0.0000 – 0.0000	<.0001
**TCD/AC (3th pct)**			
a	31.9001	28.1809 – 35.6192	<.0001
b	-2.2270	-2.6940 – -1.7600	<.0001
c	0.0810	0.0618 – 0.1002	<.0001
d	-0.0010	-0.0012 – -0.0007	<.0001
**TCD/AC (97th pct)**			
a	48.6127	43.1549 – 54.0704	<.0001
b	-3.7614	-4.4600 – -3.0629	<.0001
c	0.1347	0.1055 – 0.1638	<.0001
d	-0.0015	-0.0019 – -0.0011	<.0001
**HC/AC (3th pct)**			
a	12.9014	11.3303 – 14.4725	<.0001
b	-0.2144	-0.4006 – -0.0281	0.0241
c	0.0071	-0.0001 – 0.0143	0.0522
d	-0.0001	-0.0002 – 0.0000	0.0185
**HC/AC (97th pct)**			
a	20.7330	18.3564 – 23.1095	<.0001
b	-0.8811	-1.1565 – -0.6056	<.0001
c	0.0325	0.0222 – 0.0429	<.0001
d	-0.0004	-0.0006 – -0.0003	<.0001

Table S3: Results of logistic regression analysis for the gestational-age depended association between sonographic parameters and small gestational age. Model fit was performed for each parameter separately, including gestational age, sonographic parameter and an interaction term between gestational age and parameter. AC = abdominal circumference, BPD = biparietal diameter, CI = confidence interval, FL = femur length, HC = head circumference, TCD = transcerebellar diameter.

Sonographic parameter	Gestational age in weeks	Odds ratio	95% CI	*p* value
**HC**				
	14+0 – 18+6	0.977	0.963 – 0.991	0.0013
	19+0 – 23+6	0.965	0.957 – 0.974	<.0001
	24+0 – 28+6	0.952	0.944 – 0.961	<.0001
	29+0 – 33+6	0.931	0.920 – 0.941	<.0001
	34+0 – 38+6	0.888	0.868 – 0.908	<.0001
	>=39+0	0.897	0.802 – 1.004	0.0595
**BPD**				
	14+0 – 18+6	0.933	0.888 – 0.979	0.0052
	19+0 – 23+6	0.893	0.869 – 0.917	<.0001
	24+0 – 28+6	0.858	0.833 – 0.883	<.0001
	29+0 – 33+6	0.801	0.772 – 0.831	<.0001
	34+0 – 38+6	0.707	0.662 – 0.754	<.0001
	>=39+0	0.709	0.492 – 1.022	0.0649
**AC**				
	14+0 – 18+6	0.968	0.953 – 0.983	<.0001
	19+0 – 23+6	0.935	0.926 – 0.943	<.0001
	24+0 – 28+6	0.927	0.916 – 0.938	<.0001
	29+0 – 33+6	0.920	0.909 – 0.931	<.0001
	34+0 – 38+6	0.885	0.866 – 0.904	<.0001
	>=39+0	0.849	0.692 – 1.042	0.1167
**FL**				
	14+0 – 18+6	0.884	0.835 – 0.936	<.0001
	19+0 – 23+6	0.805	0.776 – 0.834	<.0001
	24+0 – 28+6	0.773	0.740 – 0.807	<.0001
	29+0 – 33+6	0.735	0.701 – 0.771	<.0001
	34+0 – 38+6	0.700	0.651 – 0.753	<.0001
	>=39+0	0.314	0.091 – 1.087	0.0675
**TCD**				
	14+0 – 18+6	0.873	0.772 – 0.986	0.0284
	19+0 – 23+6	0.861	0.811 – 0.913	<.0001
	24+0 – 28+6	0.854	0.811 – 0.898	<.0001
	29+0 – 33+6	0.920	0.886 – 0.955	<.0001
	34+0 – 38+6	0.891	0.850 – 0.933	<.0001
	>=39+0	0.806	0.606 – 1.073	0.1401
**FL/AC**				
	14+0 – 18+6	0.839	0.739 – 0.953	0.0068
	19+0 – 23+6	1.053	0.979 – 1.133	0.1639
	24+0 – 28+6	1.247	1.122 – 1.385	<.0001
	29+0 – 33+6	1.396	1.255 – 1.553	<.0001
	34+0 – 38+6	1.977	1.675 – 2.333	<.0001
	>=39+0	2.973	0.886 – 9.973	0.0776
**TCD/AC**				
	14+0 – 18+6	1.668	1,.72 – 2.028	<.0001

	19+0 – 23+6	2.249	1.999 – 2.531	<.0001
	24+0 – 28+6	2.421	2.065 – 2.838	<.0001
	29+0 – 33+6	1.962	1.732 – 2.224	<.0001
	34+0 – 38+6	1.600	1.394 – 1.836	<.0001
	>=39+0	1.252	0.713 – 2.199	0.4342
**HC/AC**				
	14+0 – 18+6	1.069	1.033 – 1.106	0.0001
	19+0 – 23+6	1.125	1.105 – 1.144	<.0001
	24+0 – 28+6	1.171	1.139 – 1.203	<.0001
	29+0 – 33+6	1.155	1.125 – 1.185	<.0001
	34+0 – 38+6	1.172	1.129 – 1.215	<.0001
	>=39+0	1.184	0.972 – 1.443	0.0941

Table S4: Results of logistic regression analysis for the gestational-age depended association between sonographic parameters and fetal growth restriction. Model fit was performed for each parameter separately, including gestational age, sonographic parameter and an interaction term between gestational age and parameter. AC = abdominal circumference, BPD = biparietal diameter, CI = confidence interval, FL = femur length, HC = head circumference, TCD = transcerebellar diameter.

Sonographic parameter	Gestational age in weeks	Odds ratio	95% CI	*p* value
**HC**				
	14+0 – 18+6	0.960	0.915 – 1.007	0.0912
	19+0 – 23+6	0.956	0.935 – 0.977	<.0001
	24+0 – 28+6	0.921	0.904 – 0.938	<.0001
	29+0 – 33+6	0.908	0.890 – 0.926	<.0001
	34+0 – 38+6	0.852	0.818 – 0.887	<.0001
	>=39+0	0.679	0.091 – 5.061	0.7057
**BPD**				
	14+0 – 18+6	0.906	0.774 – 1.060	0.2172
	19+0 – 23+6	0.837	0.778 – 0.901	<.0001
	24+0 – 28+6	0.775	0.732 – 0.821	<.0001
	29+0 – 33+6	0.757	0.713 – 0.804	<.0001
	34+0 – 38+6	0.626	0.558 – 0.703	<.0001
	>=39+0	0.038	NA	0.8967
**AC**				
	14+0 – 18+6	0.951	0.902 – 1.002	0.0614
	19+0 – 23+6	0.893	0.869 – 0.918	<.0001
	24+0 – 28+6	0.886	0.864 – 0.909	<.0001
	29+0 – 33+6	0.890	0.869 – 0.911	<.0001
	34+0 – 38+6	0.837	0.798 – 0.878	<.0001
	>=39+0	0.684	NA	0.9619
**FL**				
	14+0 – 18+6	0.822	0.676 – 0.999	0.0488
	19+0 – 23+6	0.694	0.625 – 0.770	<.0001
	24+0 – 28+6	0.663	0.608 – 0.724	<.0001
	29+0 – 33+6	0.654	0.601 – 0.713	<.0001
	34+0 – 38+6	0.579	0.503 – 0.666	<.0001
	>=39+0	0.005	NA	0.8686
**TCD**				
	14+0 – 18+6	0.865	0.595 – 1.259	0.4494
	19+0 – 23+6	0.906	0.784 – 1.047	0.1816
	24+0 – 28+6	0.756	0.690 – 0.828	<.0001
	29+0 – 33+6	0.908	0.854 – 0.966	0.0023
	34+0 – 38+6	0.874	0.812 – 0.940	0.0003
	>=39+0	1.133	0.632 – 2.030	0.6755
**FL/AC**				
	14+0 – 18+6	0.753	0.506 – 1.121	0.1625
	19+0 – 23+6	1.052	0.876 – 1.265	0.5853
	24+0 – 28+6	1.231	1.036 – 1.463	0.0183
	29+0 – 33+6	1.361	1.149 – 1.612	0.0004
	34+0 – 38+6	2.209	1.736 – 2.811	<.0001
	>=39+0	NA	NA	0.9151
**TCD/AC**				
	14+0 – 18+6	2.165	1.271 – 3.686	0.0045

	19+0 – 23+6	3.327	2.581 – 4.288	<.0001
	24+0 – 28+6	3.173	2.474 – 4.070	<.0001
	29+0 – 33+6	2.511	2.053 – 3.072	<.0001
	34+0 – 38+6	2.000	1.612 – 2.482	<.0001
	>=39+0	NA	NA	0.9533
**HC/AC**				
	14+0 – 18+6	1.068	0.965 – 1.183	0.2048
	19+0 – 23+6	1.193	1.147 – 1.241	<.0001
	24+0 – 28+6	1.236	1.182 – 1.293	<.0001
	29+0 – 33+6	1.192	1.145 – 1.241	<.0001
	34+0 – 38+6	1.223	1.155 – 1.295	<.0001
	>=39+0	5.035	NA	0.9379

Table S5: Evaluation of gestational age-dependent cut-off values, 10th percentile (SGA). AC = abdominal circumference, BPD = biparietal diameter, FL = femur length, GA = Gestational age, HC = head circumference, TCD = transcerebellar diameter.

Parameter	Sensitivity	Specificity	Positive predictive value	Negative predictive value	Youden Index (J)
**HC**					
**Total**	0.55	0.91	0.43	0.94	0.46
GA: 14+0– 18+6	0.35	0.90	0.18	0.95	0.25
19+0– 23+6	0.44	0.91	0.30	0.95	0.35
24+0– 28+6	0.65	0.90	0.56	0.92	0.55
29+0– 33+6	0.65	0.93	0.69	0.92	0.58
34+0– 38+6	0.64	0.90	0.65	0.90	0.54
**BPD**					
**Total**	0.51	0.88	0.35	0.93	0.39
GA: 14+0– 18+6	0.28	0.88	0.14	0.95	0.16
19+0– 23+6	0.36	0.88	0.21	0.94	0.24
24+0– 28+6	0.64	0.87	0.51	0.92	0.51
29+0– 33+6	0.65	0.87	0.55	0.91	0.52
34+0– 38+6	0.67	0.86	0.57	0.90	0.53
**AC**					
**Total**	0.74	0.90	0.48	0.96	0.64
GA: 14+0– 18+6	0.48	0.89	0.22	0.96	0.37
19+0– 23+6	0.65	0.90	0.37	0.97	0.55
24+0– 28+6	0.82	0.90	0.63	0.96	0.72
29+0– 33+6	0.83	0.89	0.65	0.96	0.72
34+0– 38+6	0.90	0.91	0.74	0.97	0.81
**FL**					
**Total**	0.65	0.90	0.44	0.95	0.55
GA: 14+0– 18+6	0.56	0.89	0.24	0.97	0.45
19+0– 23+6	0.59	0.90	0.34	0.96	0.49
24+0– 28+6	0.72	0.88	0.56	0.94	0.60
29+0– 33+6	0.70	0.90	0.62	0.93	0.60
34+0– 38+6	0.67	0.89	0.62	0.91	0.56
**TCD**					
**Total**	0.24	0.91	0.25	0.90	0.15
GA: 14+0– 18+6	0.19	0.92	0.13	0.94	0.11
19+0– 23+6	0.24	0.90	0.18	0.93	0.14
24+0– 28+6	0.30	0.91	0.39	0.86	0.21
29+0– 33+6	0.20	0.92	0.39	0.83	0.12
34+0– 38+6	0.25	0.91	0.44	0.81	0.16
**FL/AC**					
**Total**	0.40	0.78	0.19	0.91	0.18
GA: 14+0– 18+6	0.05	0.86	0.02	0.93	0.09
19+0– 23+6	0.21	0.84	0.10	0.92	0.05
24+0– 28+6	0.32	0.79	0.24	0.85	0.11
29+0– 33+6	0.60	0.58	0.26	0.86	0.18
34+0– 38+6	0.88	0.40	0.29	0.92	0.28
**TCD/AC**					
**Total**	0.23	0.94	0.33	0.90	0.17

GA:	14+0– 18+6	0.19	0.92	0.13	0.94	0.11
	19+0– 23+6	0.22	0.94	0.24	0.93	0.16
	24+0– 28+6	0.31	0.95	0.54	0.87	0.26
	29+0– 33+6	0.24	0.97	0.64	0.84	0.21
	34+0– 38+6	0.13	0.96	0.50	0.80	0.09
**HC/AC**						
**Total**		0.31	0.91	0.31	0.91	0.22
GA:	14+0– 18+6	0.19	0.90	0.11	0.94	0.09
	19+0– 23+6	0.30	0.91	0.23	0.94	0.21
	24+0– 28+6	0.36	0.91	0.44	0.87	0.27
	29+0– 33+6	0.34	0.91	0.48	0.85	0.25
	34+0– 38+6	0.27	0.94	0.55	0.82	0.21

Table S6: Evaluation of gestational age-dependent cut-off values, 3rd percentile (FGR). AC = abdominal circumference, BPD = biparietal diameter, FL = femur length, GA = Gestational age, HC = head circumference, TCD = transcerebellar diameter.

Parameter	Sensitivity	Specificity	Positive predictive value	Negative predictive value	Youden Index (J)
**HC**					
Total	0.64	0.97	0.39	0.99	0.61
GA: 14+0–18+6	0.63	0.96	0.10	1.00	0.59
19+0–23+6	0.53	0.97	0.19	0.99	0.50
24+0–28+6	0.77	0.96	0.56	0.98	0.73
29+0–33+6	0.60	0.97	0.63	0.97	0.57
34+0–38+6	0.67	0.97	0.68	0.97	0.64
**BPD**					
Total	0.45	0.98	0.40	0.98	0.43
GA: 14+0–18+6	0.38	0.97	0.08	1.00	0.35
19+0–23+6	0.37	0.98	0.19	0.99	0.35
24+0–28+6	0.59	0.97	0.58	0.97	0.56
29+0–33+6	0.41	0.99	0.78	0.96	0.40
34+0–38+6	0.42	0.98	0.71	0.95	0.40
**AC**					
Total	0.87	0.97	0.51	1.00	0.84
GA: 14+0–18+6	0.63	0.97	0.12	1.00	0.60
19+0–23+6	0.80	0.97	0.28	1.00	0.77
24+0–28+6	0.91	0.98	0.74	0.99	0.89
29+0–33+6	0.87	0.97	0.72	0.99	0.84
34+0–38+6	0.98	0.98	0.82	1.00	0.96
**FL**					
Total	0.62	0.99	0.58	0.99	0.61
GA: 14+0–18+6	0.88	0.97	0.18	1.00	0.85
19+0–23+6	0.71	0.97	0.41	1.00	0.68
24+0–28+6	0.72	0.99	0.78	0.98	0.71
29+0–33+6	0.59	0.99	0.86	0.97	0.58
34+0–38+6	0.40	1.00	1.00	0.94	0.40
**TCD**					
Total	0.20	0.97	0.17	0.98	0.17
GA: 14+0–18+6	0.25	0.97	0.06	0.99	0.22
19+0–23+6	0.25	0.97	0.09	0.99	0.22
24+0–28+6	0.30	0.96	0.34	0.96	0.26
29+0–33+6	0.13	0.98	0.34	0.94	0.11
34+0–38+6	0.10	0.97	0.23	0.92	0.07
**FL/AC**					
Total	1.00	0.01	0.03	0.99	0.01
GA: 14+0–18+6	0.88	0.09	0.01	0.99	-0.03
19+0–23+6	1.00	0.00	0.01	_	0.00
24+0–28+6	1.00	0.00	0.06	_	0.00
29+0–33+6	1.00	0.00	0.07	_	0.00
34+0–38+6	1.00	0.00	0.09	_	0.00

**TCD/AC**						
Total		0.30	0.97	0.24	0.98	0.27
GA:	14+0–18+6	0.13	0.97	0.03	0.99	0.10
	19+0–23+6	0.32	0.97	0.13	0.99	0.29
	24+0–28+6	0.34	0.97	0.40	0.96	0.31
	29+0–33+6	0.28	0.97	0.46	0.95	0.25
	34+0–38+6	0.25	0.98	0.43	0.93	0.23
**HC/AC**						
Total		0.13	0.99	0.25	0.97	0.12
GA:	14+0–18+6	0.00	0.98	0.00	0.99	-0.02
	19+0–23+6	0.22	0.99	0.18	0.99	0.21
	24+0–28+6	0.17	1.00	0.73	0.95	0.17
	29+0–33+6	0.07	1.00	0.83	0.93	0.07
	34+0–38+6	0.06	1.00	1.00	0.91	0.06
